# CSESA: an R package to predict *Salmonella enterica* serotype based on newly incorporated spacer pairs of CRISPR

**DOI:** 10.1186/s12859-019-2806-5

**Published:** 2019-04-27

**Authors:** Lang Yang, Xia Zhang, Yuqi Liu, Hao Li, Shaofu Qiu, Peng Li, Hongbin Song

**Affiliations:** 1 Academy of Military Medical Sciences, Beijing, China; 2 Center for Disease Control and Prevention of PLA, Beijing, China; 3 School of electronics engineering and computer science, Peking University, Beijing, China

**Keywords:** *S. enterica*, Serotyping, CSESA

## Abstract

**Background:**

*Salmonella enterica* is a major cause of bacterial food-borne disease worldwide. Immunological serotyping is the most commonly used typing method to characterize *S. enterica* isolates, but is time-consuming and requires expensive reagents. Here, we developed an R package CSESA (CRISPR-based *Salmonella enterica* Serotype Analyzer) to predict the serotype based on the CRISPR loci of *S. enterica*.

**Results:**

CSESA has implemented the CRISPR typing method CLSPT and extended its coverage on diverse *S. enterica* serotypes. This package takes CRISPR sequences or the genome sequences as input and provides users with the predicted serotypes. CSESA has shown excellent performance with currently available sequences of *S. enterica*.

**Conclusions:**

CSESA is a convenient and useful tool for the prediction of *S. enterica* serotypes. The application of CSESA package can improve the efficiency of serotyping for *S. enterica* and reduce the burden of manpower resources. CSESA is freely available from CRAN at https://cran.r-project.org/web/packages/CSESA/.

## Background

*Salmonella enterica*, a gram-negative bacterium of the *Enterobacteriaceae* family, is the leading cause of bacterial food-borne disease worldwide. The infection of *S. enterica* occurs as a result of consuming contaminated food and usually causes serious gastroenteritis [1]. In order to characterize various *S. enterica* isolates, immunological serotyping has been most commonly used and provided valuable information such as pathogen sources. Immunological serotyping distinguishes isolates based on the classification of somatic (O) and flagellar (H) antigens [2]. Until now, more than 2500 *Salmonella* serotypes have been identified [3]. Despite its extensive use for almost 80 years, this method still has some drawbacks. Immunological serotyping of *S. enterica* is labor-intensive, takes at least 3 days to complete, and requires experienced laboratory staff and maintenance of over 250 typing sera and 350 different antigens [4].

To avoid these disadvantages, molecular typing methods, such as multi-locus sequence typing (MLST) [5] and pulsed-field gel electrophoresis (PFGE) [6], have been adopted to predict the serotypes of *S. enterica* isolates. However, MLST has low throughput, and PFGE is time consuming and technically demanding [7]. Other molecular typing methods also have drawbacks regarding cost, speed or robustness, and cannot be evaluated as ideal methods for serotype identification on a large scale.

The clustered regularly interspaced short palindromic repeat (CRISPR) has served as a new effective marker for subtyping of *Salmonella* [8, 9]. The two CRISPR loci possessed by *Salmonella* generally comprise 29-bp direct repeats (DRs) and 32-bp variable sequences known as “spacers” [10, 11]. A CRISPR typing method based on the whole spacer arrays in both CRISPR loci has been proposed [8]. Recently, we have established a simpler molecular typing method, named CRISPR locus spacer pair typing (CLSPT) [12]. CLSPT distinguishes *S. enterica* isolates based on the pair of newly incorporated spacers in both CRISPR loci. Further analysis showed that CLSPT types presented remarkable accordance (kappa = 0.9872, Matthew’s correlation coefficient = 0.9712) with serotypes, and had good discriminatory power (discriminatory index [DI] =0.8145) [12]. However, CLSPT was established with a strictly limited dataset and remains doubtful with a large number of newly sequenced isolates in recent years. In addition, this method involves a series of genomic analysis such as identification of the newly incorporated spacer and is inconvenient for clinical use. Here, we introduce CRISPR-based *Salmonella enterica* Serotype Analyzer (CSESA), a package with the application of CLSPT to predict serotypes of *S. enterica*. We have also extended the database of CSESA with diverse *S. enterica* isolates.

## Implementation

CSESA is implemented in R and is freely available in the Comprehensive R Archive Network (CRAN). This package provides the basic function for serotype prediction with *S. enterica* CRISPR sequences. By calling the external software BLASTN [13], CSESA is also able to retrieve the CRISPR sequences and present the predicted serotype from the genome sequence.

### Data preparation

The input data for CSESA can either be CRISPR sequence(s) or the genome sequence of a *S. enterica* isolate. CRISPR sequences can be obtained by PCR amplification and sequencing with forward primer A-F (5′-GTRGTRCGGATAATGCTGCC-3′) and reverse primer A-R (5′-CGTATTCCGGTAGATBTDGATGG-3′) for CRISPR 1 locus and primers B-F (5′-GAGCAATACYYTRATCGTTAACGCC-3′) and B-R (5′-GTTGCDATAKGTYGRTRGRATGTRG-3′) for CRISPR 2 locus [8]. Each sequence of CRISPR loci should be saved to a separate file in the FASTA format. The complete genome of a *S. enterica* isolate or its draft genome in the multi-FASTA format can also be taken as input. An example dataset is included in CSESA to illustrate the input forms.

### Identification of newly incorporated spacers

**Fig. 1:**
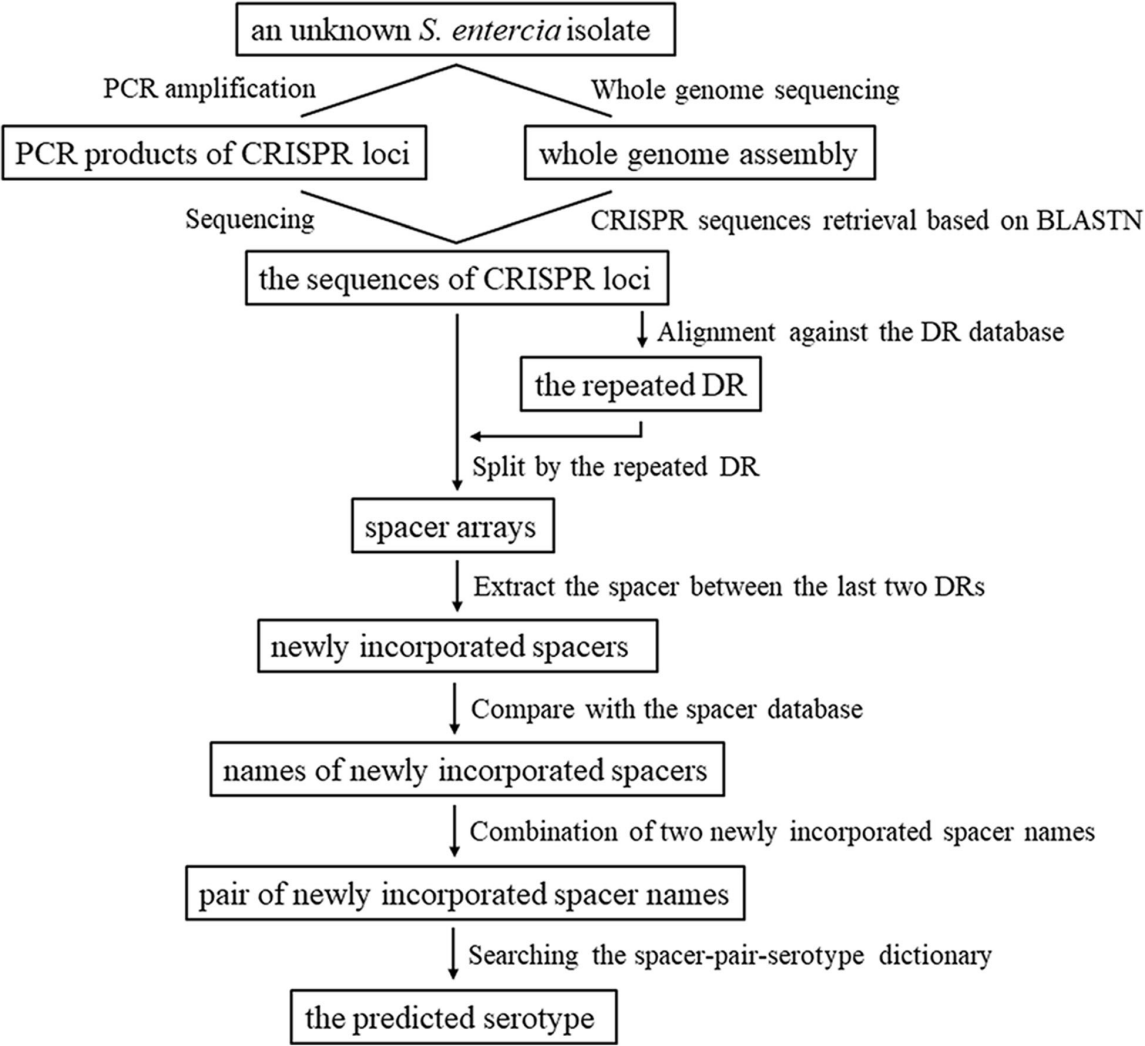
The flow diagram of CSESA package

The first step for CRISPR-based typing is to obtain the CRISPR sequences of a *S. enterica* isolate (Fig. 1). Through PCR amplification and sequencing with primers as described above, the CRISPR sequences can be acquired and directly entered into CSESA by setting “method” to “PCR” (default). Otherwise “method” should be set to “WGS” for analysis of the genome sequence. Retrieval of CRISPR sequences from the genome requires local installation of the BLASTN software. By calling BLASTN, CSESA aligns the genome against four primer sequences and locates its matched regions. The bases between primers A-F and A-R are concatenated as the sequence of CRISPR 1 locus, and those between primers B-F and B-R as the CRISPR 2 sequence.

CSESA compares the CRISPR sequences with the DR database downloaded from the Pasteur Institute’s CRISPR Database. Each CRISPR sequence is split by DRs and the last spacer between two DRs is defined as the newly incorporated one. CSESA then queried this spacer against the local database curated from Pasteur Institute’s CRISPR Database. The name of the newly incorporated spacer is obtained and used for subsequent serotype prediction.

### Serotype prediction

We have developed CLSPT for serotype prediction by establishing a local spacer-pair-serotype dictionary [12]. The dictionary is based on 537 *S. enterica* isolates from the Pasteur Institute’s CRISPR Database. The pair of newly incorporated spacers was retrieved from each isolate and mapped to the corresponding serotype, resulting in 176 correlations. In CSESA, we have extended the dictionary with 19 correlations from the collected isolates in our laboratory and 34 from the CRISPRdb (http://crispr.i2bc.paris-saclay.fr/) [14]. A total of 229 correlations are then recorded. Nine pairs of newly incorporated spacer names (namely, Ent8-EntB9, Ent4-EntB9, Heid8-STMB24, Pan1var1-BranB7, Dub1-EntB8, Chol3-TysB1, Pan1-PanB8, ConcB4var1-MonB63 and STM6-STMB24) had one-to-many correlations with serotypes and the strains sharing the same pairs of spacer names is predicted with a percentage by comparing the number of strains of the corresponding serotypes.

### Presentation of results

There are three output forms of serotype prediction. First, when the newly incorporated spacer pair from a *S. enterica* isolate find a match in the dictionary, the two spacer names and their correlated serotype are shown as the output. If this spacer pair is linked to multiple serotypes, each one with corresponding proportion will be shown. Second, only one newly incorporated spacer has been found in the dictionary, and then CSESA will list all the serotypes correlated with the pairs including this spacer. Third, when none of the newly incorporated spacers is found, a declaration of no prediction is presented. The three forms of output are shown by the example code below.

> library(CSESA).

> CSESA(“IsolateA_CRISPR_locus1.seq”, “IsolateA_CRISPR_locus2.seq”, method = “PCR”).

[1] “The newly incorporated spacer in the first CRISPR sequence: Ent8”.

[1] “The newly incorporated spacer in the second CRISPR sequence: EntB9”.

[1] “Predicted serotype(s): [Enteritidis 95%] [Nitra 2%] [Rosenberg 2%] [Blegdam 1%]”.

> CSESA(“IsolateB_genome_assembly.seq”, method = “WGS”)

[1] “The newly incorporated spacer in the first CRISPR sequence: Ago13”.

[1] “The newly incorporated spacer of the second CRISPR sequence is not available for prediction.”

[1] “Predicted serotype(s): [Agona]”.

> CSESA(“IsolateC_CRISPR_locus1.seq”, method = “PCR”)

[1] “Sorry. We did not find any corresponding serotype in the lib!”

## Results

**Fig. 2:**
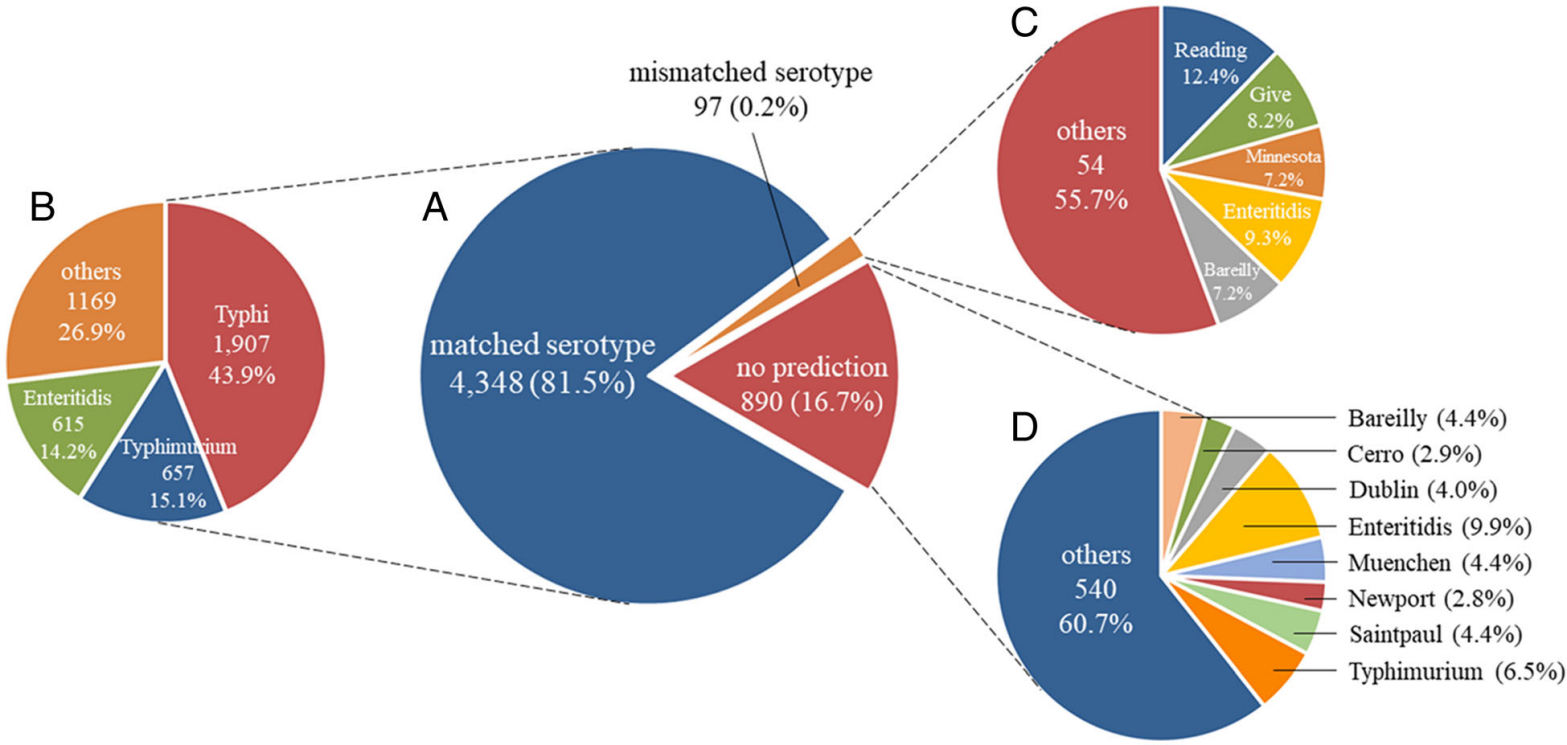
The serotype composition among three types of CSESA output with current 5335 *S. enterica* genome sequences from NCBI. **a** The distribution of three types of output from CSESA. **b**, **c** and **d** The serotype composition of matched results, mismatched results and no prediction, respectively

Genome sequences of 5335 currently available *S. enterica* isolates with determined serotypes were downloaded from the NCBI database for the test of CSESA (accessed 24th March 2018). The accuracy of CSESA was evaluated by comparing the predicted result with provided serotype of each isolate. The results indicated that 4348 of the 5335 isolates were successfully predicted with matched serotypes. Whereas 97 isolates returned mismatched serotypes and 890 isolates returned no prediction (Fig. 2).

**Fig. 3:**
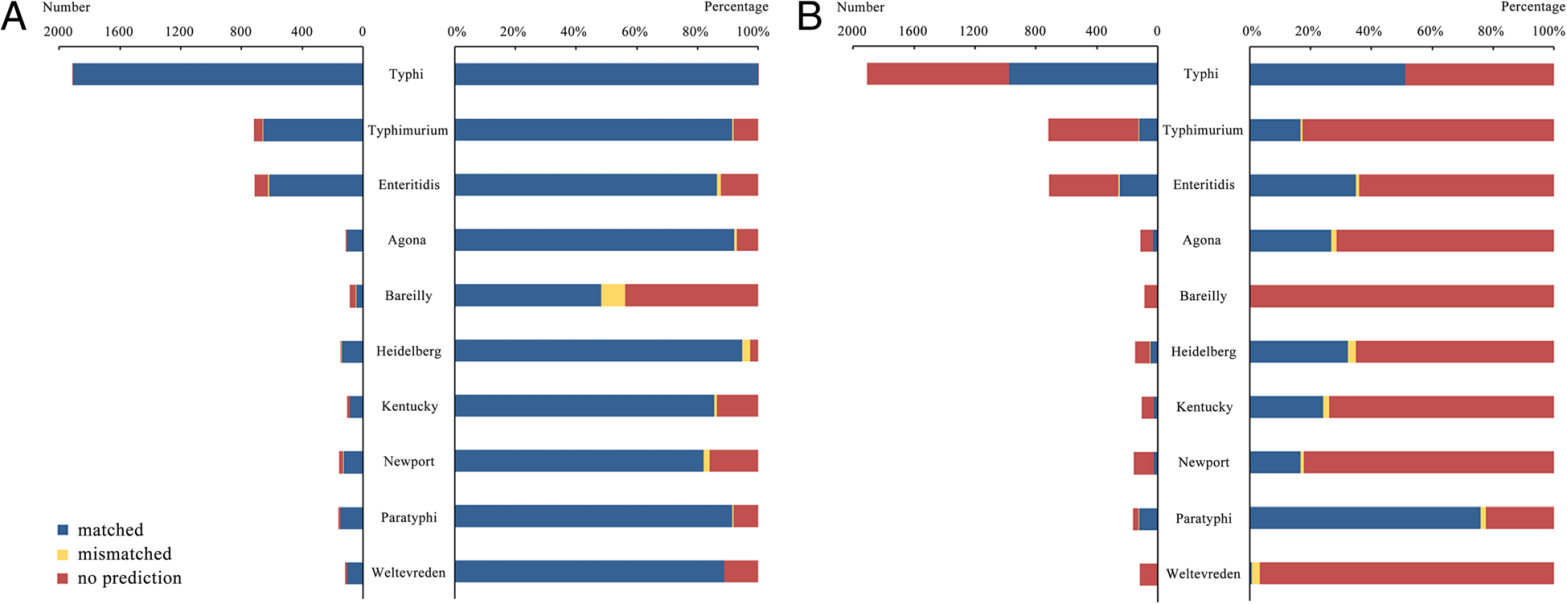
The distribution of output among common *S. enterica* serotypes from (**a**) CSESA and (**b**) CCT. The blue, yellow and red columns represent results of matched serotypes, mismatched serotypes and no prediction, respectively. The left diagram in each subfigure shows the number of results among common *S. enterica* serotypes, while the right diagram shows their proportions

CSESA has shown excellent performance on prediction with common serotypes of *S. enterica*. For the wildly distributed serotype Typhi, CSESA has successfully predicted the serotype for 99.9% (1907/1909) of the isolates. Around 90% of the isolates among other common *S. enterica* serotypes (91.4% of Typhimurium, 86.4% of Enteritidis, 92.0% of Agona, 94.6% of Heidelberg, 85.4% of Kentucky, 82.1% of Newport, 91.3 of Paratyphi and 88.8% of Weltevreden) were also correctly predicted (Fig. 3a). However, only 48.3% of the isolates of Bareilly got the matched serotype in CSESA due to the limitation of current dictionary.

We have also implemented a local perl script of the conventional CRISPR typing method [8] (referred to here as CCT) for comparison. CCT was tested with the same dataset. The results revealed that 1665 of 5335 genomes were correctly identified by CCT, which had a significantly lower accuracy of serotype prediction than CSESA (Chi-square test, *p* < 0.001). CCT showed varied performances with different *S. enterica* serotypes, and 51.0% of Typhi, 16.8% of Typhimurium, 35.0% of Enteritidis, 26.8% of Agona, 32.2% of Heidelberg, 24.3% of Kentucky, 16.7% of Newport and 75.8% of Paratyphi were correctly predicted (Fig. 3b). In addition, CCT exhibited poor performance with serotypes Bareilly and Weltevreden, and none of 89 Bareilly isolates and only one of 116 Weltevreden isolates were correctly predicited.

To Further improve CSESA’s performance in clinical situations, these correlations identified in the downloaded *S. enterica* genomes from NCBI have also been integrated into the dictionary. A total of 388 correlations covering 137 serotypes and 323 newly incorporated spacer pair polymorphisms are currently documented.

## Discussion

Although traditional immunological serotyping works as the most important method to characterize *Salmonella*, it is time- and resource-consuming and limited to well-equipped laboratories. In this study, we developed an R package CSESA to predict the serotypes of *S. enterica*. This implementation utilized the last spacers of two CRISPR loci in a *S. enterica* isolate. The utilization of CSESA requires simple PCR amplification and sequencing of the two CRISPR sequences and has remarkable effectiveness relative to the conventional CRISPR typing method. This implementation provides an ideal tool to predict *S. enterica* serotypes especially on a large scale.

CSESA has integrated the data from the Pasteur Institute’s CRISPR Database, CRISPRdb, NCBI and our local laboratory and now is able to predict 137 serotypes, covering most of the common *S. enterica* serotypes, such as Typhi, Typhimurium, Enteritidis and Paratyphi. However, the performance of CSESA is still limited by current data. Some pairs of newly incorporated spacers have mapped to different serotypes due to limited information of specific correlation. It is imperative that continuing efforts should be made to extend the spacer-pair-serotype dictionary. With increasingly available information of spacer and serotype correlations, we hope this dictionary could cover a majority of the > 2500 *Salmonella* serotypes or at least the typically encountered ones, which will make considerable savings on the time and manpower costs.

## Conclusion

In this paper, we have introduced an R package CSESA which is aimed to predict serotypes of *S. enterica*. This package has applied our previously established CRISPR typing method CLSPT and extended the serotype-targeted dictionary. CSESA showed remarkable performance on serotype prediction with the majority of *S. enterica*. This implementation supports the direct input of CRISPR sequences or the genome sequences, and is accessible to users less familiar with genomic analysis.

## Availability and requirements

**Project name:** CSESA.

**Project home page:** https://cran.r-project.org/web/packages/CSESA/index.html

**Operating system(s):** Platform independent.

**Programming language:** R.

**Other requirements:** For full functionality, the BLAST software must have been installed.

**License:** GPL-2 | GPL-3.

**Any restrictions to use by non-academics:** According to GPL.

## Abbreviations

BLAST: Basic local alignment search tool
CCT: Conventional CRISPR typing
CLSPT: CRISPR locus spacer pair typing
CSESA: CRISPR-based *Salmonella enterica* Serotype Analyzer
PCR: Polymerase chain reaction
